# Chronic pain-like responses revealed by allodynia and hyperalgesia in the ant *Lasius niger*

**DOI:** 10.1186/s12915-026-02784-4

**Published:** 2026-11-06

**Authors:** Moana Vorjans, Meghan Barrett, Tomer Joseph Czaczkes

**Affiliations:** 1 Animal Cognition and Ecology Laboratory, Department of Biology, Chemistry, Pharmacy, Freie Universität Berlin, Berlin, 14195 Germany; 2 Animal Comparative Economics Laboratory, Faculty of Biology and Preclinical Medicine, University of Regensburg, Regensburg, 93053 Germany; 3 Department of Biology, Indiana University Indianapolis, Indianapolis, IN 46202 USA

**Keywords:** Pain, Allodynia, Hyperalgesia, Arthropod, Amputation, Burn

## Abstract

**Background:**

Allodynia (pain in response to normally non-noxious stimuli) and hyperalgesia (increased sensitivity to painful stimuli) are well-established indicators of pain sensitization after tissue damage in vertebrates. These phenomena may also offer valuable insights into nociceptive processing in invertebrates. We assessed behavioural evidence for allodynia and hyperalgesia following two types of peripheral injury (tarsus amputation or burning) and sham treatment in black garden ants (*Lasius niger*, Hymenoptera: Formicidae). At 20 min, 24 h, and 96 h post-anaesthesia, a ramping hot plate assay with sublethal, but increasingly noxious, temperatures was used to assess allodynia and hyperalgesia. Locomotor speed and proportion of time spent moving were quantified to detect increased responsiveness at normally non-noxious temperatures (allodynia) or exaggerated responses at higher temperatures (hyperalgesia).

**Results:**

We observed an earlier onset of responsiveness in ants with tarsus amputations and burns compared to controls, consistent with allodynia, which was stronger and more consistent in tarsus-amputated ants and at later time points. Evidence for hyperalgesia was limited and time-dependent: amputated ants showed a steeper response increase at higher temperatures at earlier time points, but not at 96 h. Burn-treated ants showed less consistent evidence of allodynia and no evidence of hyperalgesia.

**Conclusions:**

These findings suggest that sensitization extends beyond the acute injury phase and align with broader evidence for chronic, pain-like processing in insects. As a new injury model in insects, the burn treatment needs to be refined, or the underlying mechanisms may be different to those involved in classic, amputation-induced sensitization.

**Supplementary Information:**

The online version contains supplementary material available at https://doi.org/10.1186/s12915-026-02784-4.

## Background

The question of pain, as distinct from nociception, in non-human animals such as insects has long been a topic of scientific interest and ethical concern [1–7]. Nociception, the ability to detect harmful stimuli and respond reflexively, is well documented across animal taxa [8–12] and is increasingly understood in insects. For instance, noxious stimuli are perceived by nociceptors, which are specialized sensory neurons located at the insect periphery, which fire when potentially damaging stimuli are sensed [13]. This information is then transmitted to the central nervous system, including the ventral nerve cord and higher-order sensory integration centres in the protocerebrum of the brain [10, 14–17]. Ascending nociceptive pathways mediate both rapid escape and sustained avoidance [18] while descending control contributes to the processing of nociceptive signals [19, 20]. Both pathways can be targeted with analgesics to modulate nocifensive responses [21, 22].

While nociception is increasingly well studied, pain (defined as the affective experience that accompanies noxious stimulation or tissue damage), is much harder to assess in non-human animals given its inherent subjectivity [23–25]. Accordingly, most research on the possibility of pain in non-human animals focuses on neurobiological or behavioural proxies that increase the likelihood that pain, and not mere nociception, exists [11, 26, 27] without ever definitively proving pain [23]. Recent syntheses using a common framework for evaluating the plausibility of sentience in invertebrates [26] suggest that pain in at least some orders of adult insects is plausible, particularly in the Diptera and Blattodea, with substantial evidence also found in the Hymenoptera [4].

Chronic changes in behaviour as a result of noxious stimulation, which often require central processing in higher-order brain centres and descending control of locomotor behaviour [10, 18, 21], are expected to provide stronger evidence for the plausibility of pain-like states than acute responses that may be mediated reflexively [28, 29]. Two promising avenues for investigating chronic pain-like states in insects are neuropathic allodynia and hyperalgesia, which represent paradigms for the study of chronic pain-like states without proving pain itself. In mammals, allodynia is defined as a condition where non-noxious stimuli, such as mild heat or touch, are perceived as painful after nerve injury [30, 31]. Hyperalgesia, by contrast, is defined as a heightened response to stimuli that are normally painful, and is linked to injury-induced sensitization and lowered nociceptor thresholds [30].

Both forms of pathological sensitization have received minimal empirical attention in insects to date. Following peripheral nerve injury via leg amputation, adult fruit flies developed a delayed but long-lasting (21 days after injury) sensitized escape response to previously innocuous heat (38 °C), consistent with thermal allodynia. The injury triggered caspase-dependent apoptosis of gamma-aminobutyric acid (GABA)-ergic interneurons in the ventral nerve cord leading to central disinhibition of the escape circuit. Crucially, the response was not mediated by a local reflex: head ganglia were still required for an escape response to be mounted by the animal (decapitated flies with an intact ventral nerve cord could not complete escape responses upon leg stimulation [10]). In fruit fly larvae, different isoforms of the noxious heat sensing ion channel, transient receptor potential ankyrin 1 (TRPA1), contribute to allodynia. The isoform TRPA1-D is active in peripheral neurons and is responsible for acute nociception. However, after chronic tissue damage caused by ultraviolet (UV)-C light, the larvae began to show rolling behaviour at lower temperatures, indicating signs of allodynia. This response is mediated by a different isoform, TRPA1-C, which is active mainly in neurons in the brain rather than in the periphery. When TRPA1-C is disrupted, allodynia disappears, while normal nociceptive responses at the periphery remain intact [32].

Hyperalgesia in response to normally noxious thermal stimulation has been observed in fruit fly larvae following UV-induced epidermal damage [33]: following irradiation, the proportion of larvae responding rapidly to a 45 °C stimulus increased significantly and average withdrawal latency (time to initiate a corkscrew-like escape rolling response) also decreased. This sensitization persisted for about 24 h before returning to baseline. Neural research suggests that hyperalgesia and allodynia arise through distinct signalling pathways and may or may not involve neural apoptosis. Nevertheless, both conditions result from neural sensitization: damage to tissue and nerves can enhance the activity of ascending neural circuits while reducing the efficacy of descending inhibitory pathways, thereby lowering nociceptor thresholds and contributing to both allodynia and hyperalgesia [22, 33].

To date, these pathological, chronic pain-like states have only been studied in the dipteran, *Drosophila melanogaster* [10, 32, 33]). No studies have examined allodynia or hyperalgesia in Hymenoptera, despite their complex cognition, ecological and agricultural importance, and the substantial evidence for sentience in adult Hymenoptera (while acknowledging there are still serious evidence gaps [4]). Adult Hymenoptera possess thermal nociception [34, 35] and higher-order sensory integration centres in the brain [36–38]. They can perform motivational trade-offs using noxious stimuli [39, 40], site-specific grooming responses to thermal damage [41], and flexible learning and memory in response to noxious stimulation [42–45]. These data suggest that at least some adult Hymenoptera may possess the neural architecture that supports allodynia and/or hyperalgesia responses, warranting further behavioural investigation into these states in Hymenoptera. While *D. melanogaster* research has been pivotal in identifying the mechanisms of pain sensitization in insects, extending investigation to a broader phylogenetic range is important to establish whether these phenomena are widely conserved in insects (of which there are approximately 5.5 million species [46]) or specific to *D. melanogaster* [4]. Hymenoptera are a particularly compelling target: ants have been conceptualized as superorganisms in which individuals function similarly to cells or tissues of a single body [47]. Consequently, eusocial insects might be expected to show reduced pain-like responses given the well-documented role of self-sacrifice in colony defence [48]. The observation of allodynia and hyperalgesia in such species would therefore be conceptually significant.

Accordingly, we investigated thermal sensitization in the black garden ant, *Lasius niger* (Hymenoptera: Formicidae). We measured behavioural responses following two types of peripheral injury: tarsus amputation (the classic injury model used with fruit flies [10]), and tarsus burning, a new thermal injury model which keeps the leg intact (unlike the amputation) but may still induce nociceptive sensitization. We also included a sham treatment to control for any effects of anaesthesia, manipulation, and assay repetition. Using the hot plate assay for escape behaviour thresholds developed in Khuong et al. (2019) [10], we quantified locomotor speed and activity over time to assess whether injured ants exhibit increased responsiveness to previously non-noxious temperatures (allodynia) or exaggerated responses to noxious temperatures (hyperalgesia). Escape from noxious heat is an adaptive behaviour to promote survival in ants [49], and similar heat-evoked responses in *D. melanogaster* and *Periplaneta americana* (Blattodea: Blattidae), including jumping, rapid withdrawal, and increased walking velocity, are interpreted as nocifensive behaviours [10, 12, 18, 19]. We hypothesized that both peripheral injury models (tarsus amputations and burns) would induce heightened thermal sensitivity at both innocuous and noxious temperatures, indicative of nociceptive sensitization.

## Results

To test for chronic thermal sensitization following injury, we used the black garden ant *Lasius niger*. Ants were subjected to one of three treatments targeting the middle left (mesothoracic) tarsus: sham handling, tarsus amputation, or thermal burn injury. Thermal allodynia and hyperalgesia were assessed at 20 min, 24 h, and 96 h post-anaesthesia using a ramping hot plate assay (adapted from Khuong et al. 2019 [10]; see Fig. 6 for an overview of the experimental design). Full experimental details are provided in the “Methods” section.

### Thermal preference

To confirm that the temperatures used in the chronic thermal sensitization assay to test for hyperalgesia (see the “Allodynia and hyperalgesia in the chronic thermal sensitization assay” section) are aversive to ants, we conducted a two-choice thermal preference assay, offering ants a choice between room temperature and either 32 °C or 38 °C (see the “Methods” section; Fig. 5).

**Fig. 1:**
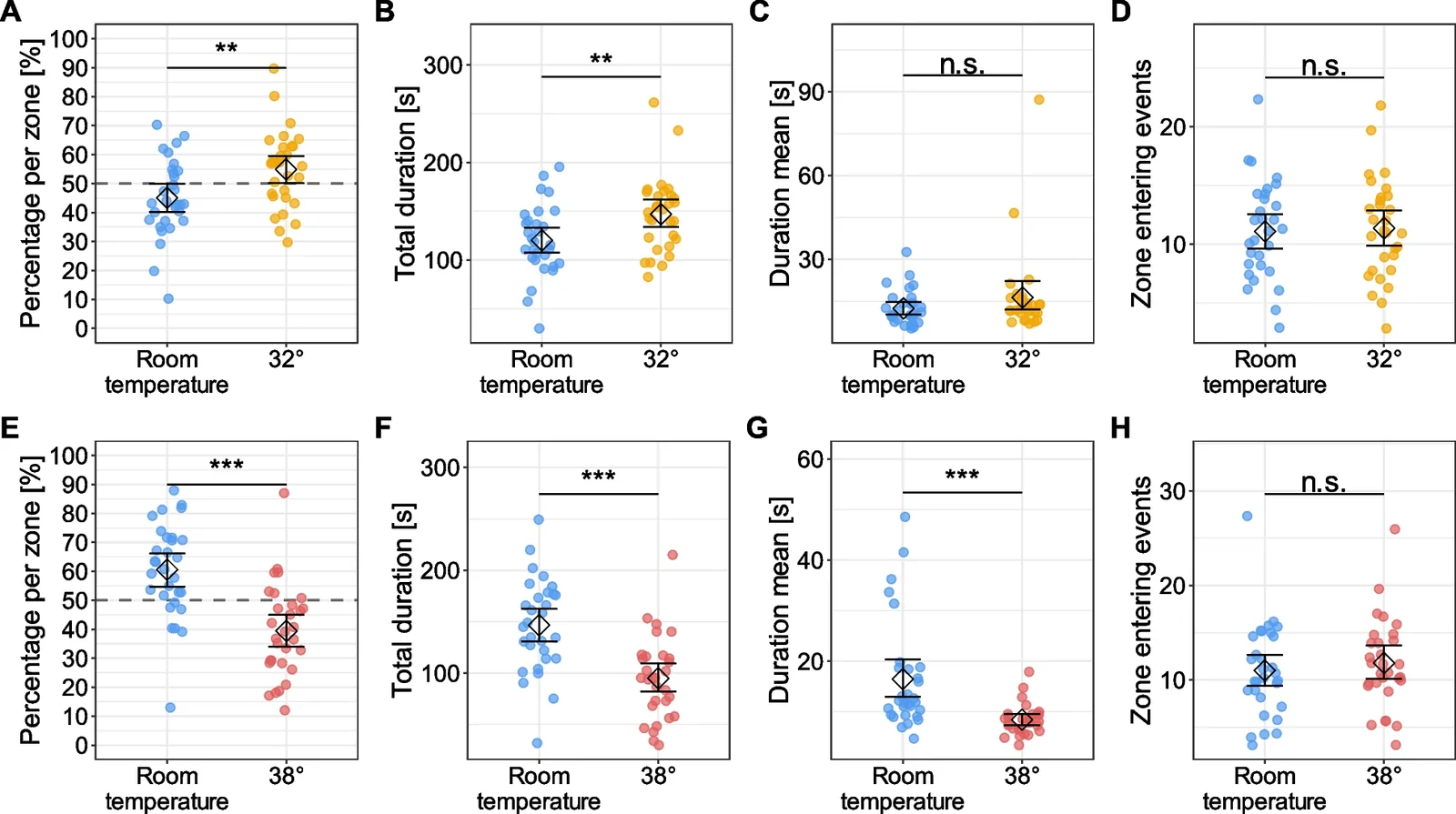
Ants prefer 32 °C but avoid 38 °C in thermal preference assay. Ants were given a choice between room temperature (~ 26 °C) and either 32 °C (**A**–**D**) or 38 °C (**E**–**H**) in an arena divided into a room-temperature zone, a heated zone, and a narrow mixed-temperature transition zone (*n* = 30 per assay); the transition zone was excluded from analysis. **A**, **E** Percentage of time spent in each zone. **B**, **F** Total duration spent in each zone. **C**, **G** Mean duration per zone visit. **D**, **H** Zone entering events. Individual data points, means (diamonds), and 95% confidence intervals are shown. ***p < 0.001, **p < 0.01, ns = not significant

Ants given a choice between room temperature and 32 °C spent significantly more time at 32 °C (~ 55%) than at room temperature (~ 45%) (LM, *p* = 0.0063), and total duration was likewise significantly greater at 32 °C than at room temperature (LM, *p* = 0.0081). Neither mean duration nor the total number of zone entries differed significantly between room temperature and 32 °C (LMM, *p* = 0.19 and *p* = 0.61, respectively) (Fig. 1A–D, Additional file 1: Table S1).

In contrast, ants given a choice between room temperature and 38 °C spent significantly more time at room temperature (~ 61%) than at 38 °C (~ 39%), both as percentage of time (LM, *p* < 0.0001) and total duration (LM, *p* < 0.0001). Ants entered the room-temperature and 38 °C zones with similar frequency (LMM, *p* = 0.15), but consistently left the 38 °C zone sooner, resulting in significantly shorter mean bout durations there than at room temperature (LMM, *p* = 0.0001) (Fig. 1E–H, Additional file 1: Table S1).

Together, these results show that intact ants prefer 32 °C over room temperature but avoid 38 °C in favour of room temperature, indicating that 38 °C is aversive/nociceptive, and that the aversive/nociceptive threshold lies between these two temperatures.

### Validation of the burn treatment

To validate that the burn treatment successfully induced tissue damage, locomotor behaviour was compared at room temperature between a control, sham-treated group and the burn-treated group, recorded at 20 min and 24 h post-anaesthesia.

**Fig. 2:**
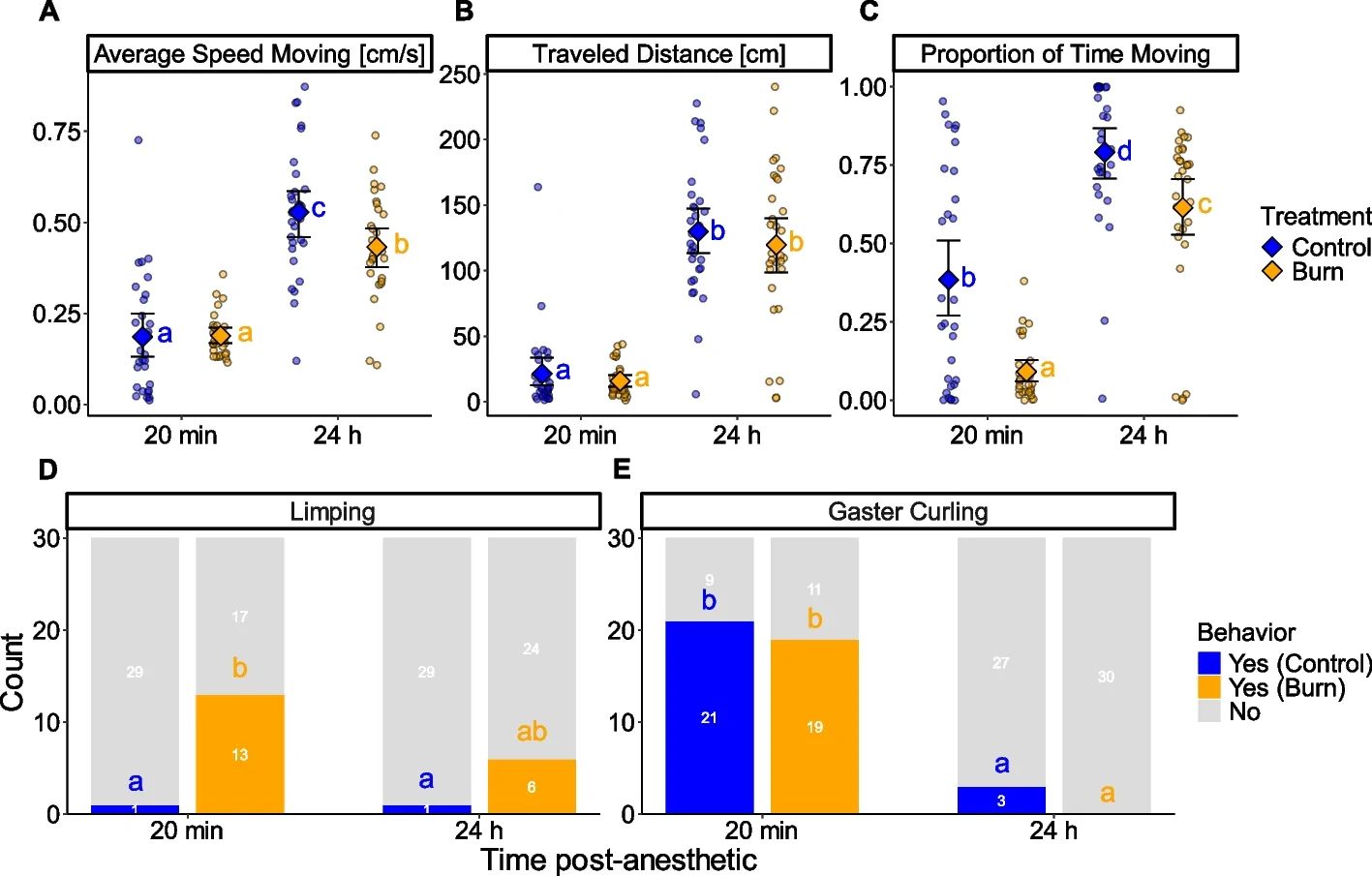
Validation of the burn treatment. **A**–**C** Burned ants moved significantly slower and spent less time moving than control ants 24 h post-anaesthesia. Movement was reduced at 20 min post-anaesthesia compared to 24 h, demonstrating that recovery was still ongoing. Individual data points are shown as dots, while diamonds represent group means; error bars denote 95% bootstrapped confidence intervals. **D**–**E** Limping was observed at higher rates in burned ants than control ants at 20 min post-anaesthesia and persisted in a subset at 24 h. Gaster curling was observed at similar rates between controlled and burned ants and was mostly observed 20 min post-anaesthesia. For each group, the number of individuals displaying the behaviour (yes/no) is shown. Different letters indicate significant differences between groups at *p* < 0.05. *N* = 30 ants per treatment per time point

For all locomotor metrics, there was a significant effect of recovery time following anaesthesia: ants exhibited higher walking speeds, travelled longer distances, and spent more time moving 24 h post-anaesthesia compared to 20 min post-anaesthesia (Wilcoxon rank-sum tests or beta regressions, all *p* < 0.001). This pattern was consistent across both treatment groups (Fig. 2A–C).

While there were no significant differences between control and burn ants at either time point for distance travelled (*p* > 0.29, Fig. 2B), average speed while moving at 24 h was significantly lower in burn-treated ants than controls (*p* = 0.038; Fig. 2A). Further, proportion of time spent moving was influenced by both treatment and recovery time. At 20 min post-anaesthesia, burn-treated ants spent only 9% of their time moving compared to 38% in control ants (beta regression, *p* < 0.001); by 24 h, burned ants spent 61% of their time moving compared to 79% in control ants (*p* < 0.001; Fig. 2C). These results suggest a lasting impact of the burn treatment on activity levels. Additionally, the significant increase in the proportion of time spent moving for all ants from 20 min to 24 h suggests that anaesthesia recovery was still ongoing at the 20-min mark (Fig. 2A–C, Additional file 1: Tables S2–S3).

In addition to locomotor metrics, we assessed the prevalence of two specific behaviours, limping (defined as “the action of walking with difficulty, typically because of a damaged leg or foot”) and gaster curling (for a video of these behaviours, see [50]), that may indicate discomfort, stress or “pain” in ants. Limping was rare in control ants at both time points (3%, 1 individual) but was observed in 43% of the burn-treated ants at 20 min post-anaesthesia and remained present in 20% of burn-treated ants at 24 h (Fig. 2D). This difference between control and burn was statistically significant at 20 min (Fisher’s exact test: *p* < 0.001) but not at 24 h (*p* = 0.1028), and limping did not vary significantly over time within either treatment (control: *p* = 1; burn: *p* = 0.095; Additional file 1: Table S4). This suggests that limping is specifically associated with the burn injury rather than the anaesthetic itself.

In contrast, gaster curling was mostly observed 20 min post-anaesthesia in both treatments (Fig. 2E) and was completely absent from burn-treated ants at 24 h. Statistically, curling did not differ between treatments at either time point (20 min: *p* = 0.785; 24 h: *p* = 0.24), but both groups showed a highly significant decrease in curling from 20 min to 24 h (control: *p* < 0.001; burn: *p* < 0.001; Additional file 1: Table S4). These findings suggest that gaster curling is likely a response to isoflurane exposure and/or handling stress, rather than to the burn treatment.

### Allodynia and hyperalgesia in the chronic thermal sensitization assay

Amputation had a less pronounced effect than burning on both movement speed and activity shortly after treatment (Additional file 2: Fig. S1–S2). At 20 min post-anaesthetic, amputated ants moved significantly faster and spent more time moving than burned ants at most temperatures ≥ 30 °C (movement speed: difference = 0.08–0.56, *p* < 0.05; proportion of time moving: difference = 0.05–0.11, *p* < 0.01), and their locomotor behaviour was generally similar to that of control ants, suggesting that this injury model has comparatively smaller impacts on locomotor ability.

We next assessed the noxious thermal threshold temperature (at which Δ speed or time spent moving significantly differed from zero) in control, amputation, and burn ants in the ramping assay at 20 min, 24 h, and 96 h. If these indicators occurred at a lower temperature in injured ants relative to controls at 96 h, this would suggest chronic allodynia.

**Fig. 3:**
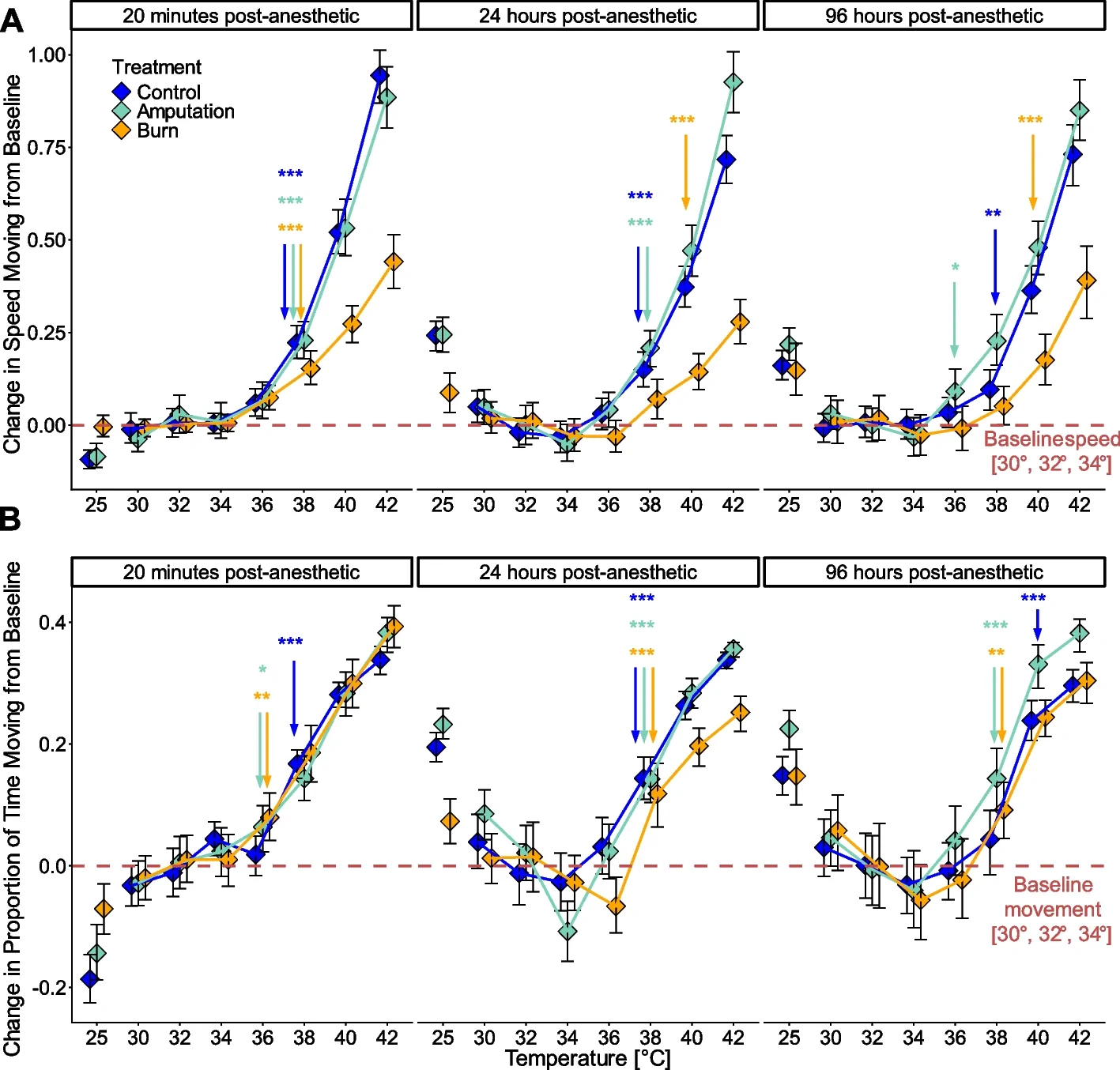
Evidence for acute and chronic lowering of thermal response thresholds following injury. **A** Change in average movement speed relative to baseline. Ninety-six hours after anaesthesia, amputated ants exhibited a lower noxious thermal threshold compared to control ants, a sign of allodynia, while burn-treated ants showed a delayed response compared to control and amputated ants. **B** Change in proportion of time spent moving relative to baseline. Injured ants increased their proportion of time spent moving at lower temperatures than controls at 20 min and 96 h post-anaesthesia, suggesting chronic allodynia. At 24 h, all groups responded similarly at 38 °C. Red dotted lines show baselines (average from 30 to 34 °C); coloured arrows mark significant increases for each group. Diamonds represent group means; error bars denote 95% bootstrapped confidence intervals. ***p < 0.001, **p < 0.01, *p < 0.05, ns = not significant. Sample sizes of 70–100/treatment, dependent on survival (see Additional file 1: Table S7). Note: isoflurane recovery was not complete after 20 min (see the “Discussion” section), so data from this time point should be used with caution

A sharp increase in locomotor speed was observed in all treatments between 36 °C and 40 °C at 20 min, 24 h, and 96 h post-anaesthesia (Fig. 3A). At 20 min post-anaesthesia, all groups had a noxious thermal threshold of 38 °C (GLMM, all p ≤ 0.001), the same pattern as observed in the baseline trial (Additional file 2: Fig. S3). At 24 h, control and amputation ants still had a noxious thermal threshold of 38 °C (*p* < 0.0001), while the noxious thermal threshold for burn-treated ants was now 40 °C (*p* < 0.0001). At 96 h, the noxious thermal threshold was now 36 °C in amputated ants, consistent with allodynia (GLMM, *p* = 0.01), while it was still 38 °C in control ants (*p* = 0.009) and 40 °C in burned ants (*p* < 0.0001) (Fig. 3A, Additional file 1: Table S5).

Like movement speed, a sharp increase in proportion of time moving was observed in all treatments between 36 °C and 40 °C (Fig. 3B). At 20 min, both amputated and burn ants showed a significant increase in responsiveness beginning at 36 °C (GLMM, all *p* < 0.05), while control ants exhibited a significant increase only from 38 °C onwards (*p* < 0.0001). However, by 24 h, all groups exhibited increased movement at 38 °C (GLMM, all *p* < 0.0001). Yet, by 96 h post-anaesthesia, both amputated and burn-treated ants yet again showed significant increases in activity at a lower temperature (38 °C; both *p* < 0.007) compared to controls (40 °C; *p* < 0.0001), indicative of chronic allodynia (Fig. 3B, Additional file 1: Table S6).

We next assessed whether the intensity of the response to noxious temperatures differed by treatment (hyperalgesia). To do this, slopes for each treatment-time combination at noxious temperatures (38–42 °C) were compared for both movement speed and proportion of time spent moving. A stronger temperature response (i.e. higher slope) in a treatment group compared to the control may indicate hyperalgesia.

**Fig. 4:**
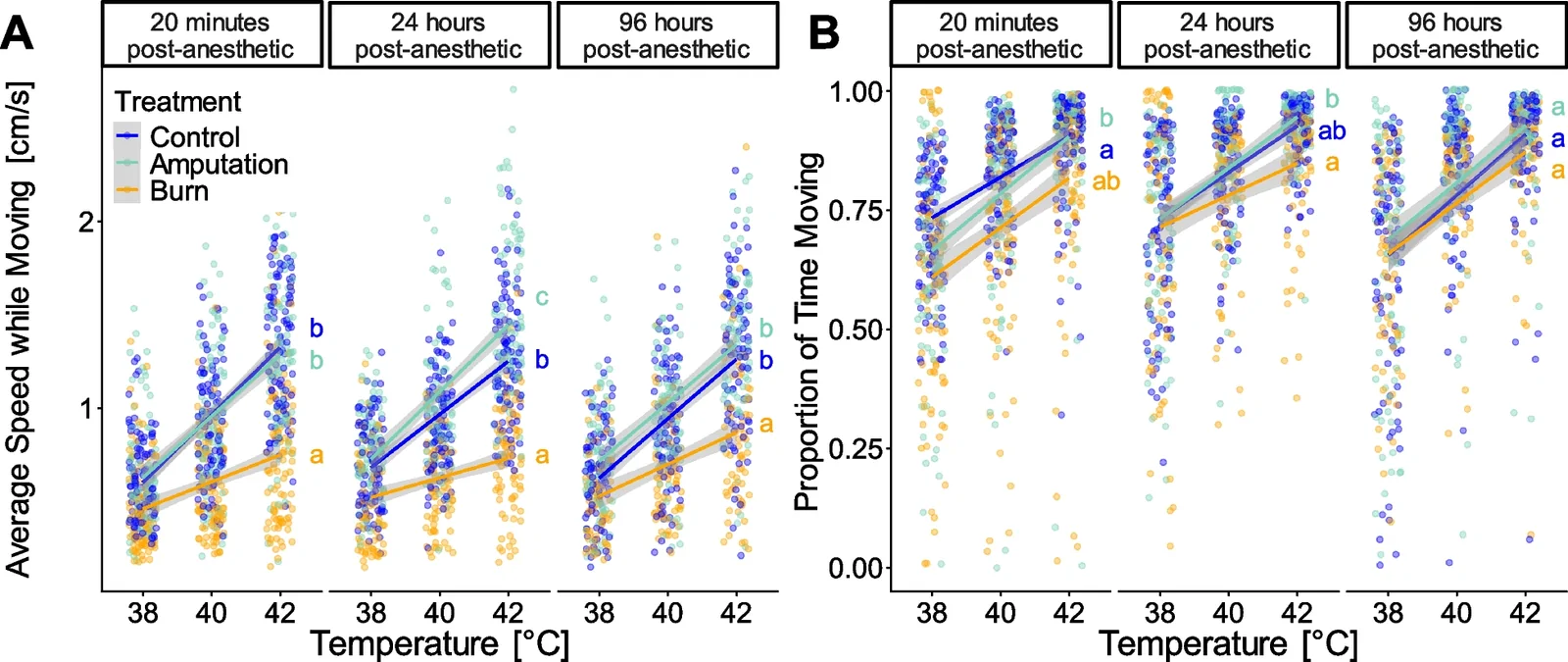
Some evidence for amputation-induced steepening of thermal response slopes at noxious temperatures. **A** Effect of noxious temperatures (38–42 °C) on movement speed across treatments and timepoints. At 24 h post-anaesthetic, amputated ants showed the steepest temperature-response slope to noxious temperatures, while burned ants consistently had the lowest slope. **B** Effect of noxious temperatures (38–42 °C) on proportion of time moving across treatments and timepoints. Effects were weaker in this behavioural metric. Across time points, amputated ants showed slightly higher slopes than control ants, which was significant at 20 min post-anaesthesia. Points represent individual observations. Linear models were fitted for each treatment, with 95% confidence intervals shown. Treatments not sharing a letter are significantly different from each other at the respective temperature (*p* < 0.05). Sample sizes of 70–100/treatment, dependent on survival (see Additional file 1: Table S7). Note: isoflurane recovery was not complete after 20 min (see the “Discussion” section), so data from this time point should be used with caution

At both 20 min and 96 h post-anaesthesia, amputation and control treatments did not differ significantly (GLMM, all *p* > 0.55) (Fig. 4A). However, at 24 h, all pairwise comparisons showed significant differences (GLMM, all *p* ≤ 0.011), indicating divergent slopes between treatments. Amputation showed the steepest temperature response, indicative of hyperalgesia. By contrast, burn ants consistently showed the lowest slopes, indicating a reduced responsiveness to noxious temperatures (GLMM, all *p* < 0.0001) (Fig. 4A, Additional file 1: Table S8).

We performed the same analysis for proportion of time spent moving (Fig. 4B). At 20 min post-anaesthesia, the amputation group showed a significantly higher slope than control ants (GLMM, *p* = 0.0338), indicative of hyperalgesia, while all other comparisons were non-significant (all *p* > 0.33). By 24 h, burn-treated ants again exhibited a significantly weaker temperature response compared to amputation (GLMM, *p* = 0.0201). In this case no significant differences were observed between amputation and control (*p* = 0.73) or burn and control (*p* = 0.12). Finally, by 96 h post-anaesthesia, no significant differences were detected among any treatment groups (all *p* > 0.53) (Fig. 4B, Additional file 1: Table S9). Across time points, amputation generally showed slightly higher estimated values than the other treatments.

### Survival in response to injuries for the chronic thermal sensitization assay

Ant survival was monitored daily for 96 h following anaesthesia and injury (or sham treatment) during the chronic thermal sensitization assay. A log-rank test showed significantly reduced survival in the burn group compared to both amputation (chi-squared = 6.4, df = 1, *p* = 0.01) and control groups (chi-squared = 8.9, df = 1, *p* = 0.003). No significant difference was found between amputation and control groups (chi-squared = 0.2, df = 1, *p* = 0.70).

The experiment began with 100 ants per treatment group at time 0. After 96 h, 87 control ants, 85 amputated ants, and 70 burn-treated ants were still alive (see Additional file 1: Tables S10–S11, Additional file 2: Fig. S4). This increased mortality in the burn-treated group suggests this injury model may result in significantly more internal damage than the amputation, despite being a less visually apparent injury externally.

## Discussion

This study assessed potential chronic pain-like responses (hyperalgesia and allodynia) in ants following mechanical (amputation) and thermal (burn) tarsal injuries. The findings suggest evidence for thermal allodynia and some evidence for hyperalgesia in ants, particularly following amputation, concurring with prior literature using the amputation model in *D. melanogaster* [10].

Allodynia was evident in amputated ants at 96 h following the injury and was corroborated by both an increased movement speed and proportion of time spent moving at a lower temperature compared to controls. Earlier time points (20 min, 24 h) did not differ consistently from controls. This pattern mirrors amputation injury models in *D. melanogaster*, where allodynia emerges after 5–7 days and persists for weeks [10], suggesting conserved mechanism for neuropathic-like pain across insects. The delayed onset may reflect time-dependent neural plasticity, including apoptosis of GABAergic interneurons and altered cholinergic signalling as reported in *D. melanogaster* [10]. Hyperalgesia was also evident in our amputated ants. At 24 h, amputated ants showed steeper speed increases with increasing temperatures than controls at noxious temperatures, consistent with thermal hyperalgesia. Proportion-time-moving data also indicated hyperalgesia in amputated ants at 20 min. However, these effects were not consistent across timepoints, with no evidence of hyperalgesia at 96 h. The early response at 20 min should be interpreted with caution, as ants may not have fully recovered from anaesthesia at this timepoint, potentially confounding behavioural responses. Further investigation controlling for anaesthesia recovery time would help clarify the temporal dynamics of hyperalgesia following amputation.

Average walking speed likely reflects response intensity, whereas proportion of time moving captures movement initiation. Combined, these measures provide a more complete behavioural assessment of putative, chronic pain-like states.

A further consideration is that tarsal amputation involves not only tissue injury but also sensory ablation, as the tarsus bears multiple kinds of sensory neurons [51, 52]. This raises the possibility that altered behavioural responses reflect sensory loss rather than nociceptive sensitization. However, this confound is difficult to avoid entirely in any peripheral injury model, since peripheral tissues damaged by injury typically also contain non-nociceptive sensory innervation. Similar limitations apply to peripheral injury models used to study pain-like states in both vertebrate and invertebrate species. Here, sensory loss would be expected to reduce, not enhance, responsiveness, the opposite of what we observed, as amputated ants never reacted later or less than control ants. The heightened responses we observed in amputated ants therefore more likely reflect sensitization. Further, as with prior experiments of this nature in *D. melanogaster* [53], the ants retained five other limbs with intact sensory neurons; this suggests that ants are unlikely to have lost any sensory abilities through damage only to the middle, left limb.

In contrast to the relatively straightforward results produced by the amputation injury model, burn-treated ants were inconsistent in both allodynic and hyperalgesic responses even at 96 h post-injury. For instance, while the ‘proportion of time spent moving’ data indicated allodynia by 96 h, the increase in locomotor speed occurred at a later temperature than controls—the opposite of what would be expected under allodynia. Similarly, movement speed data at 96 h demonstrated the opposite pattern as would be predicted under hyperalgesia (i.e. less sensitivity to the previously noxious temperatures), and proportion of time spent moving data showed no difference from controls. These data could be a result of the burn treatment causing more severe damage than anticipated, especially in comparison to the amputation model: burn-treated ants had reduced survival at 96 h (70% compared to 87% for controls and 85% for amputated ants) and 20% were still visibly limping or walking with an uncoordinated gait at 24 h post-injury. This raises the possibility that the thermal injury extended beyond the directly burned area, causing additional damage to more proximal leg segments [54, 55], potentially through tissue necrosis or inflammation, and thereby strongly impairing locomotion [56–58]. Slower or less movement in this group is thus likely to reflect a fundamental locomotor confound inherent to the burn treatment model rather than altered nociception.

Locomotor ability remained high in limb-amputated ants, evidenced by similar movement speeds and proportion of time spent moving when compared to controls at all time points post-injury. Insects are well-known to compensate for limb loss and preserve locomotor function [53]. In the future, limb-loss models of injury may prove best for assessing insect chronic sensitization because of the lower risk of mortality and reduced locomotor confound (as observed in our results). However, being able to compare the impacts of multiple mechanisms and severities of tissue damage on chronic sensitization may still prove valuable in future work if the burn treatment can be further refined.

Notably, in poikilotherms, locomotion strongly depends on environmental temperature [59–62]; therefore, increased locomotor speed at higher temperatures could in principle reflect elevated metabolic rates rather than aversive or nociceptive processing. In our baseline assay validation, we found that ants’ movement speed increased with temperature; however, this trend was non-linear, being flat between 25 °C and 36 °C before rising sharply at ≥ 38 °C (see Additional file 2: Fig. S3). This transition occurs near temperatures known to activate insect thermosensory TRPA channels. Hymenoptera-specific TRPA (HsTRPA) channels are activated at temperatures in the low-to-mid 30 °C range, including HsTRPA from *Atta texana* (> 33 °C [63]) and the *Apis mellifera* ortholog (AmHsTRPA; ~ 34 °C [34]). *D. melanogaster* larvae exhibit nocifensive responses above 39 °C [13, 64]. Importantly, sensory thresholds are expected to precede lethal thermal limits (~ 45 °C in ants [65, 66]), as the adaptive function of nociception is to promote avoidance before irreversible tissue damage occurs.

While the metabolic rate confound limits the interpretation of baseline movement speed as a direct indicator of pain or nociception in ants, the effect also applies equally across treatments; therefore, all relative comparisons between treatment groups in our study (used to support allodynia or hyperalgesia) remain valid. We therefore interpret higher hot plate temperatures as increasingly aversive, without claiming that the increased locomotor responses observed reflect pain in the strict neurophysiological sense. Further, movement speed data were corroborated by proportion of time spent moving data—which is likely to be less impacted by any metabolic confound.

A limitation of this study is that the same individuals were tested repeatedly across temperature conditions (as in prior research on allodynia and hyperalgesia in both vertebrates and invertebrates [10, 67]), which may have influenced behaviour through habituation or cumulative stress. Absolute values should therefore be interpreted with caution, as measured responses may partly reflect prior exposure history. However, since the primary aim was to compare responses across treatments, and all individuals underwent the same testing protocol, any carry-over effects are likely evenly distributed across conditions, leaving relative differences between treatments valid. A fully independent design would have required a substantially larger number of animals be subjected to permanent tissue damage intended to cause chronic ‘pain-like’ states and was therefore considered ethically unjustifiable given the success and widespread use of repeated testing across temperature conditions in prior research approaches.

A related limitation concerns the handling of repeated measures in the thermal sensitization assay. Ants were tested in groups of five at three time points. While groups could be matched across different time points, individual ants could not be identified within groups because they were not individually marked. Pseudo-IDs (see the “Methods” section) therefore link repeated observations from the same group position but cannot capture consistent differences between individual ants over time. The pseudo-ID analysis produced essentially the same results for allodynia as the original analysis, whereas results for hyperalgesia differed slightly. We therefore consider the allodynia result robust to this limitation and interpret the hyperalgesia result more cautiously.

Altogether, our results support both allodynia and hyperalgesia in amputated ants. This injury was mechanical, yet the heightened sensitivity emerged in response to thermal stimuli. This cross-modality effect, whereby a mechanical injury influences sensitivity to a different sensory modality (thermal stimulus), suggests that prior tissue damage can enhance responsiveness to different noxious inputs, consistent with central sensitization and shared or interacting nociceptive pathways [30]. Alongside aforementioned results in *D. melanogaster* adults that suggest the central nervous system is essential for behavioural responses to chronic pain-like states [10], research in tobacco hornworm larvae also shows hyperalgesia following tissue damage is mediated by central nervous system changes (involving N-methyl-D-aspartate receptors and hyperpolarization-activated cyclic nucleotide-gated channels in central ganglia and dorsal nerve branches [68, 69]). These studies, when combined with our own results, highlight that allodynia and hyperalgesia are not unique to vertebrates but are broadly conserved among, minimally, many holometabolous insects.

Pharmacological validation is a critical next step: if analgesics (nonsteroidal anti-inflammatory drugs, GABAergic agonists) restore control-like behaviour in injured ants, it would indicate that observed behaviours are modifiable states rather than hardwired reflexes [22, 70–74]; however, locomotor confounds will need to be carefully considered and controlled for with any analgesics, as some can have sedative side effects. Preference-based paradigms, including conditioned place preference or avoidance, could further reveal cognitive integration and motivational prioritization, extending beyond reflexive nociception [11, 75, 76]. Future priorities include metabolomic and molecular assays (e.g. fat utilization, octopamine levels) to monitor stress physiology [77, 78]. Greater understanding of how a variety of injury models beyond amputations may affect chronic, pain-like responses would also be valuable for understanding the roles of complex damage-induced pathways related to apoptosis, inflammation, or biomechanical limitation. Finally, combining multiple behavioural metrics, including walking velocity [18], leg withdrawal (mirroring paw withdrawal responses in vertebrates, i.e. the Hargreaves test [79]), wall following or repeated contact with or active probing of the arena boundary for an exit as temperatures increase, could improve detection of pain-like states.

## Conclusions

There is a tremendous amount left to learn about chronic, pain-like responses, and the neurobiology that supports them, in insects. While far from conclusive, our results make the hypothesis that chronic, pain-like responses are present throughout the Insecta more likely.

## Methods

### Colony maintenance

Ten colonies of the black garden ant *Lasius niger* [80], collected in Regensburg, Germany (48°59′42.08″ N, 12°5′26.92″ E) in April and June 2024, and in Berlin, Germany (52°27′57.1″ N, 13°18′03.9″E) in May 2026, were used in this study. Each colony was housed in a separate plastic foraging box measuring 32.7 × 22.7 × 11.1 cm, with a layer of plaster of Paris on the bottom. The lids of the boxes contained a meshcovered opening to allow ventilation. To prevent escape, the inner walls were coated with a mixture of talc powder and ethanol (20 g of talc and 100 ml of 20% ethanol). Each foraging box contained a plaster nest covered with cardboard to provide shelter. The colonies were maintained on a 12:12 h light:dark cycle at 22–26 °C and 45–55% relative humidity. The ants were provided with water, a 0.5 M sucrose solution (Südzucker AG, Mannheim, Germany) and chopped frozen cockroaches as a protein source ad libitum.

### Anaesthesia protocol

Isoflurane (CAS 26675–46-7; Forene, Abbott GmbH, Wiesbaden, Germany), a halogenated ether recommended for use with invertebrates by veterinarians [81], anaesthesia was carried out following MacMillan et al. (2017) [82] under a fume hood. Briefly, ten forager ants were placed in a 50 ml airtight induction chamber (50 ml SuperClear Centrifuge Tubes, Labcon), stoppered with a cotton ball. A pipette was used to inject 50 µl of liquid isoflurane into the lower part of the cotton ball, and the tube was quickly sealed. Ants were exposed to the anaesthetic for 3 min. The exposure time of 3 min was chosen based on the results of a previous experiment (Vorjans and Czaczkes, *in prep*) and allowed for sufficient handling time to induce the injury as well as high long-term survival.

### Thermal preference assay

**Fig. 5:**
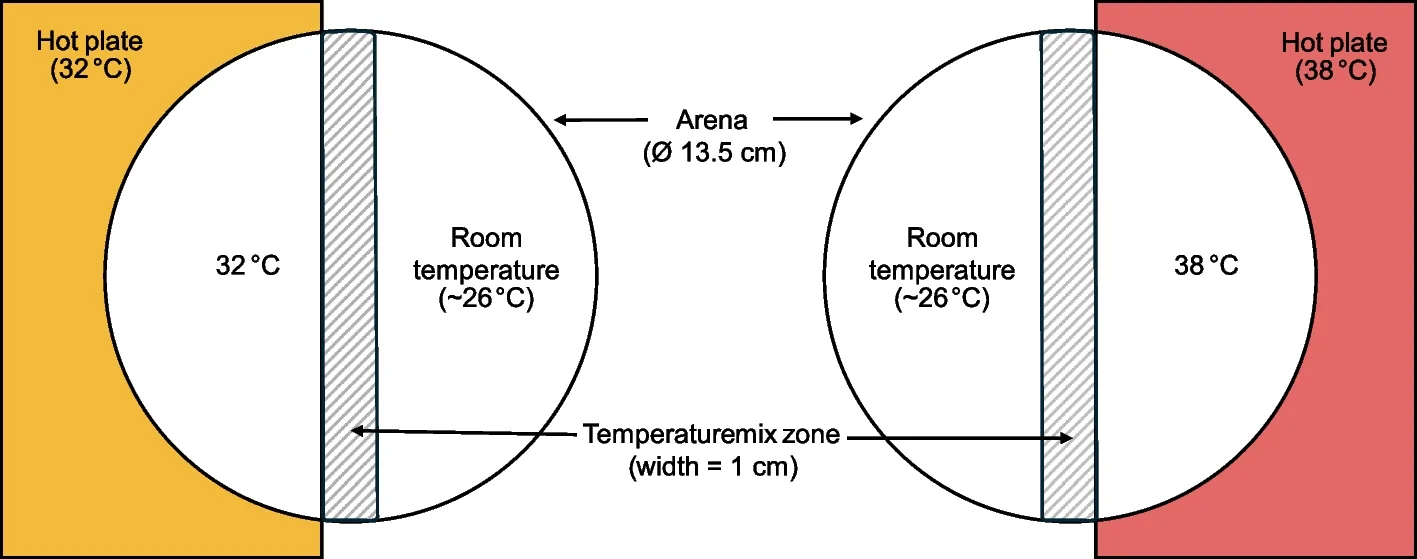
Thermal preference assay setup. Individual ants were placed in an arena divided into three zones: room temperature, a heated zone (32 °C or 38 °C), and a narrow mixed-temperature transition zone. After a 10-min acclimation period, ant position was recorded for 5 min. The position of the heated side was alternated between trials

To confirm that the temperatures used in the chronic thermal sensitization assay (see the "Thermal sensitization assay for allodynia and hyperalgesia" section) are aversive to ants, we conducted a two-choice thermal preference assay (Fig. 5). Ants were placed individually in an arena (Ø 13.5 cm) divided into three zones: 45% at room temperature (~ 26 °C), 45% on a heated substrate (Techne DRI-BLOCK DB 3), and a narrow (~ 10%) mixed-temperature transition zone in the centre. This assay was run in two separate setups: one offering a choice between room temperature and 32 °C (*n* = 30), and one offering a choice between room temperature and 38 °C (*n* = 30), reflecting the boundary of the noxious range (38–42 °C) used in the ramping assay to test for hyperalgesia. Surface temperatures were validated with an IR-thermometer (Voltcraft IR 500-12D). Walls of the arena were covered with a mixture of talc and 20% ethanol to prevent escape. Following a 10-min acclimation period, ant position was tracked for 5 min using BORIS (v. 9.11.0 [83]). Position was defined as the zone containing the majority of the ant’s body. Hot-plate side was alternated across trials to control for positional bias. The assay was conducted on three independent days with ants from five colonies.

### Main experimental setup

A hot plate (Biometra TB1 Thermoblock) was used for all experiments. Two arenas (floorless plastic rings, ∅ 5 cm, height 1.5 cm, covered with a mixture of talc and 20% ethanol, plastic lid on top to prevent escape) were placed on filter paper on top of the hot plate. The filter papers were changed after each round to ensure that no residual pheromone trails from previous ants could affect the behaviour of those in subsequent trials. Ants were video recorded (resolution: 1920 × 1080 pixels, frame rate: 25 frames per second) from above using a Raspberry Pi 4 B (Python script by Henrique Galante) and an HQ camera. Videos were analysed (frame rate of analysis: 25 frames per second) using AnimalTA [84].

#### Validation of the burn treatment

This experiment was used to assess whether controlled heat damage to the leg of the ant could be induced using a soldering iron. It was conducted on 4 days using ants from three colonies. Thirty ants per treatment (control or burn) were anaesthetized in groups of ten, as previously described. Control ants were handled on the middle left tarsus as a sham procedure, while burn-treated ants were touched on the middle left tarsus for 10 s with a soldering iron heated to 150 °C (BLANKO DIGITAL temperature-controlled soldering station, ZD-937 ELECTRONIC TOOL). Ants were then allowed to recover for 20 min before being placed in the arena at room temperature and video recorded for 5 min. Following recordings, ants were removed from the arena and kept in separate Petri dishes containing water ad libitum, drops of 0.5 M sucrose solution and a cover. They were recorded again for 5 min after 24 h. The following movement and behavioural metrics were extracted using AnimalTA software [84, 85] or manual observations:

- Average speed while moving (cm/s): Mean speed calculated only during frames where the animal was considered to be moving (when speed exceeded the movement threshold of 0.1 cm/s).
- Proportion of time moving: The fraction of total video time during which the subject was considered as moving (above movement threshold of 0.1 cm/s).
- Total distance travelled (cm): The cumulative distance covered by the ant throughout the video.
- Limping: Binary score (present/absent) and frequency based on visible, asymmetric gait during movement (not conducted blind to treatment; manual scoring).
- Gaster curling: Binary score (present/absent) and frequency recorded when the ant curled its gaster underneath its body [86], a behaviour potentially indicative of discomfort or distress [49] (not conducted blind to treatment; manual scoring).

#### Thermal sensitization assay for allodynia and hyperalgesia

**Fig. 6:**
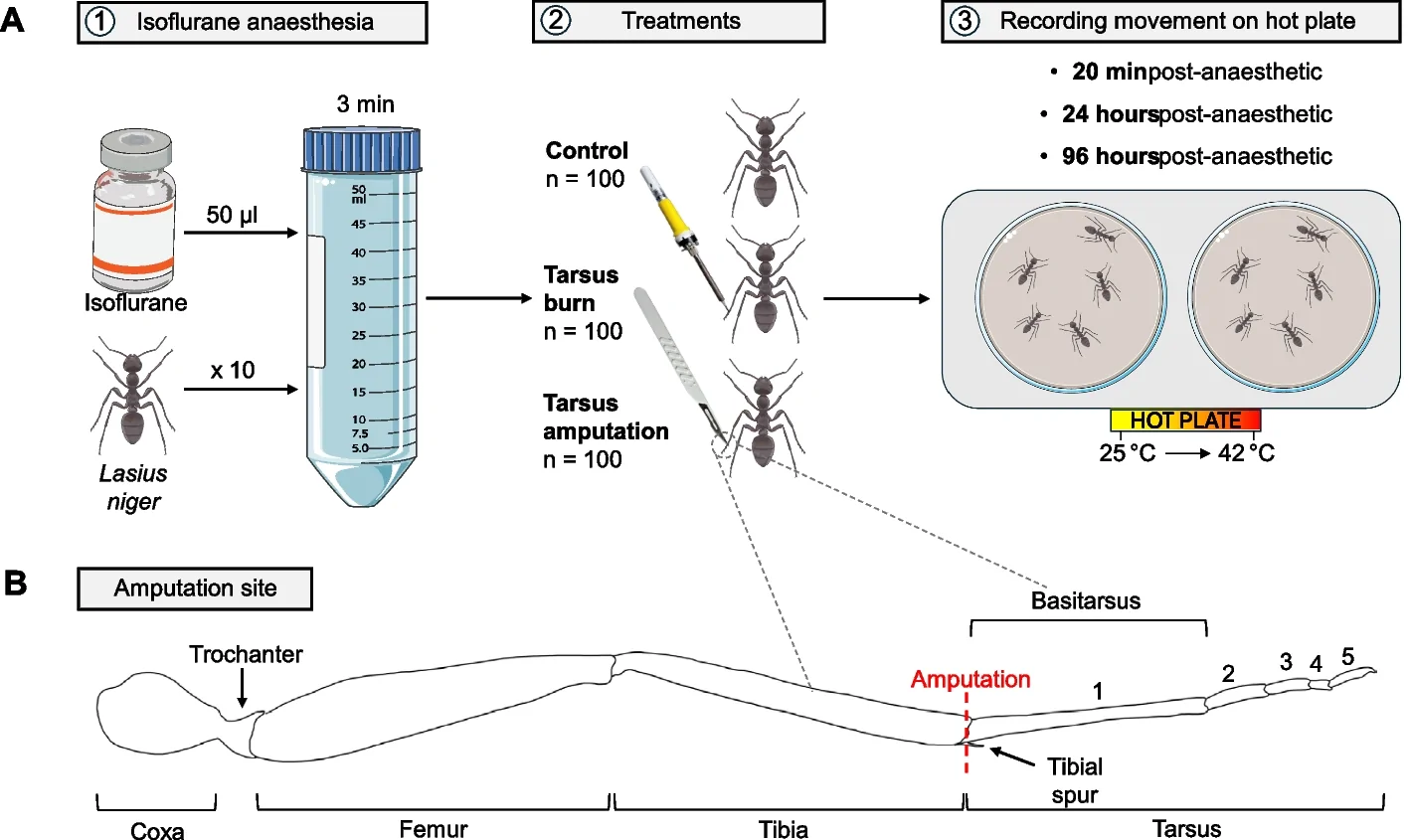
Overview of the thermal sensitization assay for allodynia and hyperalgesia. **A** Ants were anaesthetized with 1 µl/mL isoflurane for 3 min in an airtight, 50 mL induction chamber and then assigned to one of three treatment groups: control, tarsus amputation or tarsus burn (*n* = 100 per group). Movement was recorded during a ramping hot plate assay at temperatures ranging from 25 °C to 42 °C, at three time points: 20 min, 24 h, and 96 h post-anaesthesia. Movement was tracked using AnimalTA software. **B** Schematic illustration of the mesothoracic leg. The red dashed line indicates the amputation site at the tibia–basitarsus joint, resulting in removal of all tarsomeres. Illustration created using icons from BioIcons (Pictograms: https://bioicons.com/ (accessed: 20.04.2026); vaccine bottle and ant icons by DBCLS https://togotv.dbcls.jp/en/pics.html, licensed under CC-BY 4.0 https://creativecommons.org/licenses/by/4.0/ Falcon tube and Petri dish icons by Servier https://smart.servier.com/, licensed under CC-BY 3.0 https://creativecommons.org/licenses/by/3.0/ Soldering iron and scalpel photographs taken by the authors). Ant leg adapted from Frank et al. (2024) [87]

Methods for the peripheral mechanical injury (i.e. amputation) and chronic thermal sensitization paradigm were adapted from Khuong et al. (2019) [10]. Ten ants were first anaesthetized with isoflurane as previously described. They were then subjected to one of three treatments targeting the middle left tarsus: control (sham) treatment, tarsus amputation with a sterile scalpel at the tibia-basitarsus joint, resulting in removal of all tarsomeres (see Fig. 6B), or tarsus burning with a 150 °C soldering iron for 10 s (as previously described). After 20 min, 24 h, and 96 h of anaesthesia recovery, ants were recorded on the hot plate at 25 °C (acclimation period), 30 °C, 32 °C, 34 °C, 36 °C, 38 °C, 40 °C, and 42 °C (Fig. 6A). Surface temperatures were validated with an IR-thermometer (Voltcraft IR 500-12D) across the full range of experimental temperatures. Each temperature was held constant for 2 min. Temperature rise between steps was rapid (30 s), and ants were not recorded during the temperature rise. Videos were scored for movement speed, proportion of time spent moving and travelled distance with AnimalTA. Ants were maintained in Petri dishes in groups of five between tests with water ad libitum, drops of 0.5 M sucrose solution, and a cover. Ant survival was monitored each day of the experiment (from day 1 to day 5). A total of 100 ants per treatment from four colonies were tested over 23 experimental days within a 1-month period. Unlike the *Drosophila*-based assay of Khuong et al. (2019) [10], which measures escape flight responses, this approach measures locomotor activity and is thus applicable to any legged crawling animal, that, like ants, lacks an obvious escape response, broadening the range of species in which pain-like responses can be studied.

In order to test for allodynia and identify the specific temperatures at which a significant increase in movement speed occurs for different treatments, we calculated the change in speed relative to a defined baseline. This baseline was established individually for each treatment group and time point as the average speed moving at moderate temperatures (30 °C, 32 °C, and 34 °C). This was based on pre-experiment data indicating minimal speed variation within this range (see Additional file 2: Fig. S3). The change from baseline (Δ speed moving) was calculated as:

$$\begin{aligned}\Delta\ &\text{average speed moving}\ \\&=\ \text{observed speed moving}\ -\ \text{baseline speed moving}\end{aligned}$$

This was repeated for proportion of time moving data (Δ proportion moving):

$$\begin{aligned}\Delta\ &\text{average proportion of time moving}\ \\&=\ \text{observed proportion of time moving}\ \\&-\ \text{baseline proportion of time moving}\end{aligned}$$

### Statistical analysis

The full statistical analysis results, along with the complete dataset, are available via the Open Science Framework (OSF) [50]. The data were analysed and visualized using R version 4.5.0 [88].

Thermal preference data were analysed using the lme4 package [89]. Linear mixed-effects models (LMMs) with random intercepts for colony and observation ID nested within colony were fitted for each response variable. Where variance components were estimated at zero, the random effects were dropped, yielding a linear model (LM) whose results were identical to those from the full model.

For the pre-experiment validation of the burn treatment, non-parametric comparisons were performed using the Mann–Whitney *U* test (Wilcoxon rank-sum test, base R function wilcox.test) to assess differences between the control and burn groups at each time point (20 min and 24 h post-anaesthesia), as well as changes over time within each group. The metrics analysed included speed and travelled distance. Pairwise comparisons of limping and gaster curling were performed using Fisher’s exact tests (base R function fisher.test). The proportion of time spent moving was modelled using beta regression using the betareg package [90].

For testing allodynia and hyperalgesia, generalized linear mixed-effects models (GLMMs) were fitted using the glmmTMB package [91, 92], assuming a Gaussian distribution. Colony number was included as a random effect to account for variation between colonies. Ants were tested in groups of five at three time points. Because ants were not individually marked, identity could be tracked within a time point but not between time points. To account for repeated measures, we assigned pseudo-IDs consecutively across groups (group A: 1–5, group B: 6–10, and so on), so that each ID is unique to one position within one group, and included pseudo-ID nested within colony as a random effect. Pseudo-IDs therefore link repeated observations from the same position within a group but may refer to different physical ants at different time points. This accounts for repeated measures but does not produce accurate estimates of within-ant variation.

Model assumptions were validated using the DHARMa package [93]. Estimated marginal means and trends were obtained using the emmeans package [94]. Post-hoc pairwise comparisons of both trends and estimated marginal means were conducted with Tukey and Benjamini–Hochberg adjustments for multiple comparisons. Non-significant groupings (*p* > 0.05) were visualized using compact letter displays via the cld() function from the multcompView package [95]. Graphics were generated using the ggplot2 package [96].

Survival analysis was performed and visualized using the survival and survminer packages [97, 98]. Kaplan–Meier survival curves were used to visualize differences in survival between treatments. Log-rank tests were used to compare survival between treatment groups, and chi-squared statistics were used to calculate *p*-values for overall and pairwise group comparisons.

## Supplementary Information

Additional file 1: Table S1. Thermal preference between temperature zones. Table S2. Speed and distance travelled of control and burn-treated ants. Table S3. Proportion of time spent moving of control and burn-treated ants. Table S4. Limping and gaster curling in ants. Table S5. Change in average movement speed relative to baseline. Table S6. Change in proportion of time moving relative to baseline. Table S7. Sample sizes in the chronic thermal sensitization assay. Table S8. Effect of noxious temperatures (38–42 °C) on movement speed. Table S9. Effect of noxious temperatures (38–42 °C) on proportion of time moving. Table S10. Survival probability in the chronic thermal sensitization assay. Table S11. Survival analysis.

Additional file 2: Fig. S1. Average speed while moving. Fig. S2. Proportion of time spent moving. Fig. S3. Baseline average speed while moving. Fig. S4. Survival of the ants in the chronic thermal sensitization assay.

## Data Availability

The datasets and R scripts generated and analysed during the current study are available in the OSF repository (https://doi.org/10.17605/OSF.IO/7498G).

## Abbreviations

AmHsTRPA: *Apis mellifera* Hymenoptera-specific transient receptor potential ankyrin
GABA: Gamma-aminobutyric acid
GLMM: Generalized linear mixed-effects model
HsTRPA: Hymenoptera-specific transient receptor potential ankyrin
LM: Linear model
LMM: Linear mixed-effects model
OSF: Open Science Framework
TRPA: Transient receptor potential ankyrin
UV: Ultraviolet
