# Supplementary material for: Chronic pain-like responses revealed by allodynia and hyperalgesia in the ant Lasius niger
Source: BMC Biol. 2026 Nov 6;24:235. doi: 10.1186/s12915-026-02784-4 (PMC13757002; doi:10.1186/s12915-026-02784-4)
Supplement: Supplementary file 1 [file 12915_2026_2784_MOESM1_ESM.docx]

1 Thermal preference assay

Table S1: Thermal preference between temperature zones. Ants preferred 32 °C over room temperature but avoided 38 °C, while the number of zone entries did not differ between temperature zones. Linear (mixed) models were analysed using estimated marginal means (emmeans) for pairwise comparisons between temperature zones. ***p < 0.001, **p < 0.01, *p < 0.05, ns = not significant. N=30 per temperature.

| Con-trast | Response variable | RE structure | Estimate | SE | df | t-ratio | p-value |
| --- | --- | --- | --- | --- | --- | --- | --- |
| Room temp - 32° | Percent per zone | none | -9.86 | 3.48 | 57 | -2.84 | 0.0063 |
|  | Total duration | none | -26.80 | 9.77 | 57 | -2.75 | 0.0081 |
|  | Duration mean | Id only | -3.92 | 2.93 | 29 | -1.34 | 0.1921 |
|  | Zone entering | full | -0.27 | 0.51 | 29 | -0.52 | 0.6078 |
| Room temp - 38° | Percent per zone | none | 21.20 | 4.20 | 57 | 5.04 | <0.0001 |
|  | Total duration | none | 51.60 | 11.20 | 57 | 4.61 | <0.0001 |
|  | Duration mean | Colony only | 8.05 | 1.95 | 54,1 | 4.12 | 0.0001 |
|  | Zone entering | full | -0.80 | 0.55 | 29 | -1.47 | 0.1532 |

2 Validation of the burn treatment

Table S2: Speed and distance travelled of control and burn-treated ants. Significant differences in speed and distance were found for both groups over time. Between treatments, no significant differences were found at 20 min, or in distance at 24 h, but average speed while moving differed significantly at 24 h (Wilcoxon rank-sum tests). Significance levels are indicated as *p < 0.05, ***p < 0.001, and “ns” for non-significant results. N = 30 ants per treatment per time point.

| Comparison | W statistic | *p*-value | Result |
| --- | --- | --- | --- |
| Average Speed Moving (Control: 20 min vs 24 h) | 63 | 3.409e-10 | *** |
| Average Speed Moving (Burn: 20 min vs 24 h) | 72 | 2.438e-09 | *** |
| Average Speed Moving (20 min: Control vs Burn) | 352 | 0.2927 | ns |
| Average Speed Moving (24 h: Control vs Burn) | 572 | 0.03783 | * |
| Travelled Distance (Control: 20 min vs 24 h) | 45 | 9.083e-12 | *** |
| Travelled Distance (Burn: 20 min vs 24 h) | 79 | 1.633e-09 | *** |
| Travelled Distance (20 min: Control vs Burn) | 441 | 0.9007 | ns |
| Travelled Distance (24 h: Control vs Burn) | 486 | 0.6022 | ns |

Table S3: Proportion of time spent moving of control and burn-treated ants. Proportion of time spent moving was influenced by both treatment and recovery time. At both time points, burn-treated ants spent significantly less time moving than control ants (Beta regression). Statistical significance is indicated as ***p < 0.001 and “ns” for non-significant results. Positive estimates indicate greater proportions of time moving in the first group of the contrast. N = 30 ants per treatment per time point.

| Comparison | Estimate | SE | z ratio | p-value | Result |
| --- | --- | --- | --- | --- | --- |
| Control 20 min – Burn 20 min | 0.264 | 0.048 | 5.48 | < 0.001 | *** |
| Control 20 min – Control 24 h | -0.395 | 0.052 | -7.65 | < 0.001 | *** |
| Control 20 min – Burn 24 h | -0.123 | 0.083 | -1.48 | 0.832 | ns |
| Burn 20 min – Control 24 h | -0.659 | 0.050 | -13.09 | < 0.001 | *** |
| Burn 20 min – Burn 24 h | -0.387 | 0.051 | -7.54 | < 0.001 | *** |
| Control 24 h – Burn 24 h | 0.271 | 0.049 | 5.54 | < 0.001 | *** |

Table S4: Limping and gaster curling in ants. Burn-treated ants limped significantly more than control ants at 20 min but not at 24 h. Gaster curling did not differ between treatments but decreased over time in both groups (Fisher exact test). Significance levels:  ***p < 0.001, ns = not significant. “Inf” indicates an infinite odds ratio due to zero counts. N = 30 ants per treatment per time point.

| Behaviour | Comparison | Odds Ratio | 95% CI | p-value | Result |
| --- | --- | --- | --- | --- | --- |
| Limping | Control vs Burn 20 min | 0.047 | 0.001 – 0.365 | <0.001 | *** |
| Limping | Control vs Burn 24 h | 0.142 | 0.003 – 1.294 | 0.103 | ns |
| Limping | Control 20 min vs 24 h | 1 | 0.012 – 81.119 | 1 | ns |
| Limping | Burn 20 min vs 24 h | 3.001 | 0.854 – 11.705 | 0.095 | ns |
| Curling | Control vs Burn 20 min | 1.344 | 0.404 – 4.578 | 0.785 | ns |
| Curling | Control vs Burn 24 h | Inf | 0.422 – Inf | 0.237 | ns |
| Curling | Control 20 min vs 24 h | 19.654 | 4.443 – 126.950 | <0.001 | *** |
| Curling | Burn 20 min vs 24 h | Inf | 9.945 – Inf | <0.001 | *** |

3 Allodynia and hyperalgesia in the chronic thermal sensitization assay

3.1 Allodynia

Table S5: Change in average movement speed relative to baseline. 96 hours after anaesthesia, amputated ants exhibited a lower behavioural threshold to heat, while burn-treated ants showed a delayed response compared to control and amputated ants. Estimated marginal means (EMMs) for the interaction between treatment and temperature. Pairwise comparisons were conducted to test differences from zero (= baseline). Significance levels: ***p < 0.001, **p < 0.01, *p < 0.05, ns = not significant. Sample sizes of 70-100/treatment, dependent on survival.

| Time post-anaesthetic | Tempera-ture (°C) | Treatment | Estimate | SE | z-ratio | p-value | Results |
| --- | --- | --- | --- | --- | --- | --- | --- |
| 20 min | 25 | Amputation | -0.08204 | 0.0402 | -2.042 | 0.0898 | ns |
|  | 25 | Burn | -0.01556 | 0.0396 | -0.393 | 0.8750 | ns |
|  | 25 | Control | -0.08355 | 0.0398 | -2.098 | 0.0861 | ns |
|  | 30 | Amputation | -0.02802 | 0.0397 | -0.706 | 0.7203 | ns |
|  | 30 | Burn | -0.01967 | 0.0396 | -0.496 | 0.8261 | ns |
|  | 30 | Control | -0.00778 | 0.0396 | -0.196 | 0.8750 | ns |
|  | 32 | Amputation | 0.03400 | 0.0398 | 0.854 | 0.6292 | ns |
|  | 32 | Burn | -0.00777 | 0.0396 | -0.196 | 0.8750 | ns |
|  | 32 | Control | 0.01171 | 0.0398 | 0.294 | 0.8750 | ns |
|  | 34 | Amputation | 0.02043 | 0.0398 | 0.514 | 0.8261 | ns |
|  | 34 | Burn | -0.00624 | 0.0397 | -0.157 | 0.8750 | ns |
|  | 34 | Control | 0.00807 | 0.0396 | 0.204 | 0.8750 | ns |
|  | 36 | Amputation | 0.07420 | 0.0397 | 1.870 | 0.1231 | ns |
|  | 36 | Burn | 0.06342 | 0.0396 | 1.601 | 0.1940 | ns |
|  | 36 | Control | 0.06276 | 0.0396 | 1.584 | 0.1940 | ns |
|  | 38 | Amputation | 0.23999 | 0.0396 | 6.057 | <0.0001 | *** |
|  | 38 | Burn | 0.14196 | 0.0396 | 3.583 | 0.0009 | ** |
|  | 38 | Control | 0.22579 | 0.0396 | 5.699 | <0.0001 | *** |
|  | 40 | Amputation | 0.54201 | 0.0397 | 13.657 | <0.0001 | *** |
|  | 40 | Burn | 0.26241 | 0.0396 | 6.623 | <0.0001 | *** |
|  | 40 | Control | 0.52354 | 0.0396 | 13.215 | <0.0001 | *** |
|  | 42 | Amputation | 0.89612 | 0.0396 | 22.617 | <0.0001 | *** |
|  | 42 | Burn | 0.43038 | 0.0396 | 10.862 | <0.0001 | *** |
|  | 42 | Control | 0.94716 | 0.0396 | 23.907 | <0.0001 | *** |
| 24 h | 25 | Amputation | 0.24974 | 0.0312 | 7.995 | <0.0001 | *** |
|  | 25 | Burn | 0.07941 | 0.0326 | 2.439 | 0.0321 | * |
|  | 25 | Control | 0.24387 | 0.0309 | 7.893 | <0.0001 | *** |
|  | 30 | Amputation | 0.05433 | 0.0312 | 1.739 | 0.1514 | ns |
|  | 30 | Burn | 0.01043 | 0.0326 | 0.320 | 0.8168 | ns |
|  | 30 | Control | 0.05139 | 0.0309 | 1.663 | 0.1650 | ns |
|  | 32 | Amputation | 0.00757 | 0.0312 | 0.242 | 0.8437 | ns |
|  | 32 | Burn | 0.00225 | 0.0326 | 0.069 | 0.9450 | ns |
|  | 32 | Control | -0.01837 | 0.0310 | -0.593 | 0.6324 | ns |
|  | 34 | Amputation | -0.04635 | 0.0313 | -1.479 | 0.2089 | ns |
|  | 34 | Burn | -0.03753 | 0.0326 | -1.153 | 0.3321 | ns |
|  | 34 | Control | -0.02965 | 0.0309 | -0.960 | 0.4046 | ns |
|  | 36 | Amputation | 0.04729 | 0.0312 | 1.514 | 0.2081 | ns |
|  | 36 | Burn | -0.03874 | 0.0327 | -1.185 | 0.3321 | ns |
|  | 36 | Control | 0.03265 | 0.0309 | 1.057 | 0.3671 | ns |
|  | 38 | Amputation | 0.21395 | 0.0312 | 6.849 | <0.0001 | *** |
|  | 38 | Burn | 0.06161 | 0.0326 | 1.892 | 0.1170 | ns |
|  | 38 | Control | 0.15111 | 0.0309 | 4.891 | <0.0001 | *** |
|  | 40 | Amputation | 0.47658 | 0.0312 | 15.256 | <0.0001 | *** |
|  | 40 | Burn | 0.13536 | 0.0326 | 4.157 | <0.0001 | *** |
|  | 40 | Control | 0.37407 | 0.0309 | 12.108 | <0.0001 | *** |
|  | 42 | Amputation | 0.93186 | 0.0312 | 29.831 | <0.0001 | *** |
|  | 42 | Burn | 0.27052 | 0.0326 | 8.308 | <0.0001 | *** |
|  | 42 | Control | 0.71844 | 0.0309 | 23.254 | <0.0001 | *** |
| 96 h | 25 | Amputation | 0.22605 | 0.0355 | 6.361 | <0.0001 | *** |
|  | 25 | Burn | 0.14385 | 0.0380 | 3.786 | 0.0004 | *** |
|  | 25 | Control | 0.16281 | 0.0352 | 4.622 | <0.0001 | *** |
|  | 30 | Amputation | 0.03935 | 0.0355 | 1.107 | 0.4597 | ns |
|  | 30 | Burn | 0.00402 | 0.0382 | 0.105 | 0.9345 | ns |
|  | 30 | Control | -0.00290 | 0.0352 | -0.082 | 0.9345 | ns |
|  | 32 | Amputation | 0.00958 | 0.0357 | 0.269 | 0.9008 | ns |
|  | 32 | Burn | 0.01191 | 0.0382 | 0.312 | 0.9008 | ns |
|  | 32 | Control | 0.01104 | 0.0353 | 0.312 | 0.9008 | ns |
|  | 34 | Amputation | -0.02315 | 0.0357 | -0.649 | 0.7289 | ns |
|  | 34 | Burn | -0.03179 | 0.0382 | -0.833 | 0.6072 | ns |
|  | 34 | Control | 0.00335 | 0.0352 | 0.095 | 0.9345 | ns |
|  | 36 | Amputation | 0.09939 | 0.0355 | 2.797 | 0.0103 | * |
|  | 36 | Burn | -0.01429 | 0.0382 | -0.375 | 0.9008 | ns |
|  | 36 | Control | 0.03524 | 0.0353 | 0.997 | 0.5098 | ns |
|  | 38 | Amputation | 0.23559 | 0.0355 | 6.630 | <0.0001 | *** |
|  | 38 | Burn | 0.04694 | 0.0380 | 1.236 | 0.3999 | ns |
|  | 38 | Control | 0.10073 | 0.0351 | 2.869 | 0.0090 | ** |
|  | 40 | Amputation | 0.48786 | 0.0355 | 13.729 | <0.0001 | *** |
|  | 40 | Burn | 0.17178 | 0.0380 | 4.521 | <0.0001 | *** |
|  | 40 | Control | 0.36756 | 0.0351 | 10.470 | <0.0001 | *** |
|  | 42 | Amputation | 0.85850 | 0.0357 | 24.079 | <0.0001 | *** |
|  | 42 | Burn | 0.38685 | 0.0380 | 10.183 | <0.0001 | *** |
|  | 42 | Control | 0.73582 | 0.0351 | 20.960 | <0.0001 | *** |

Table S6: Change in proportion of time moving relative to baseline. At both 20 minutes and 96 hours post-anaesthesia, injured ants (amputation, burn) showed a significant increase in proportion of time spent moving at lower temperatures than control ants, suggesting possible allodynia. At 24 hours, all groups exhibited increased movement at 38 °C. Estimated marginal means (EMMs) for the interaction between treatment and temperature. Pairwise comparisons were conducted to test differences from zero (= baseline). Significance levels: ***p < 0.001, **p < 0.01, *p < 0.05, ns = not significant. Sample sizes of 70-100/treatment, dependent on survival.

| Time post-anaesthetic | Temperature (°C) | Treatment | Estimate | SE | z-ratio | p-value | Results |
| --- | --- | --- | --- | --- | --- | --- | --- |
| 20 min | 25 | Amputation | -0.14287 | 0.0275 | -5.204 | <0.0001 | *** |
|  | 25 | Burn | -0.07136 | 0.0275 | -2.599 | 0.0172 | * |
|  | 25 | Control | -0.18644 | 0.0275 | -6.791 | <0.0001 | *** |
|  | 30 | Amputation | -0.02789 | 0.0275 | -1.016 | 0.4371 | ns |
|  | 30 | Burn | -0.02077 | 0.0275 | -0.756 | 0.5676 | ns |
|  | 30 | Control | -0.03261 | 0.0275 | -1.188 | 0.3525 | ns |
|  | 32 | Amputation | 0.00601 | 0.0275 | 0.219 | 0.8268 | ns |
|  | 32 | Burn | 0.00906 | 0.0275 | 0.330 | 0.7737 | ns |
|  | 32 | Control | -0.01154 | 0.0275 | -0.420 | 0.7707 | ns |
|  | 34 | Amputation | 0.02444 | 0.0275 | 0.890 | 0.4979 | ns |
|  | 34 | Burn | 0.00916 | 0.0275 | 0.334 | 0.7737 | ns |
|  | 34 | Control | 0.04385 | 0.0275 | 1.597 | 0.1764 | ns |
|  | 36 | Amputation | 0.06461 | 0.0275 | 2.353 | 0.0319 | * |
|  | 36 | Burn | 0.07857 | 0.0275 | 2.862 | 0.0084 | ** |
|  | 36 | Control | 0.01859 | 0.0275 | 0.677 | 0.5981 | ns |
|  | 38 | Amputation | 0.14428 | 0.0275 | 5.255 | <0.0001 | *** |
|  | 38 | Burn | 0.18462 | 0.0275 | 6.725 | <0.0001 | *** |
|  | 38 | Control | 0.16758 | 0.0275 | 6.104 | <0.0001 | *** |
|  | 40 | Amputation | 0.28423 | 0.0275 | 10.353 | <0.0001 | *** |
|  | 40 | Burn | 0.29878 | 0.0275 | 10.883 | <0.0001 | *** |
|  | 40 | Control | 0.28131 | 0.0275 | 10.246 | <0.0001 | *** |
|  | 42 | Amputation | 0.38359 | 0.0275 | 13.972 | <0.0001 | *** |
|  | 42 | Burn | 0.39230 | 0.0275 | 14.289 | <0.0001 | *** |
|  | 42 | Control | 0.33821 | 0.0275 | 12.319 | <0.0001 | *** |
| 24 h | 25 | Amputation | 0.23185 | 0.0228 | 10.161 | <0.0001 | *** |
|  | 25 | Burn | 0.07120 | 0.0239 | 2.983 | 0.0049 | ** |
|  | 25 | Control | 0.19775 | 0.0226 | 8.769 | <0.0001 | *** |
|  | 30 | Amputation | 0.08514 | 0.0228 | 3.732 | 0.0004 | *** |
|  | 30 | Burn | 0.01073 | 0.0239 | 0.450 | 0.6754 | ns |
|  | 30 | Control | 0.04170 | 0.0226 | 1.849 | 0.0967 | ns |
|  | 32 | Amputation | 0.02075 | 0.0228 | 0.909 | 0.4150 | ns |
|  | 32 | Burn | 0.01273 | 0.0239 | 0.533 | 0.6477 | ns |
|  | 32 | Control | -0.00944 | 0.0226 | -0.419 | 0.6754 | ns |
|  | 34 | Amputation | -0.10827 | 0.0229 | -4.727 | <0.0001 | *** |
|  | 34 | Burn | -0.02931 | 0.0239 | -1.228 | 0.2927 | ns |
|  | 34 | Control | -0.02386 | 0.0226 | -1.058 | 0.3663 | ns |
|  | 36 | Amputation | 0.02336 | 0.0228 | 1.024 | 0.3671 | ns |
|  | 36 | Burn | -0.06834 | 0.0239 | -2.863 | 0.0067 | ** |
|  | 36 | Control | 0.03390 | 0.0226 | 1.503 | 0.1874 | ns |
|  | 38 | Amputation | 0.14245 | 0.0228 | 6.243 | <0.0001 | *** |
|  | 38 | Burn | 0.11652 | 0.0239 | 4.881 | <0.0001 | *** |
|  | 38 | Control | 0.14663 | 0.0226 | 6.502 | <0.0001 | *** |
|  | 40 | Amputation | 0.28345 | 0.0228 | 12.423 | <0.0001 | *** |
|  | 40 | Burn | 0.19498 | 0.0239 | 8.168 | <0.0001 | *** |
|  | 40 | Control | 0.26562 | 0.0226 | 11.778 | <0.0001 | *** |
|  | 42 | Amputation | 0.35580 | 0.0228 | 15.594 | <0.0001 | *** |
|  | 42 | Burn | 0.25012 | 0.0239 | 10.478 | <0.0001 | *** |
|  | 42 | Control | 0.34111 | 0.0226 | 15.126 | <0.0001 | *** |
| 96 h | 25 | Amputation | 0.22655 | 0.0273 | 8.288 | <0.0001 | *** |
|  | 25 | Burn | 0.14398 | 0.0295 | 4.887 | <0.0001 | *** |
|  | 25 | Control | 0.15292 | 0.0270 | 5.665 | <0.0001 | *** |
|  | 30 | Amputation | 0.04775 | 0.0273 | 1.747 | 0.1290 | ns |
|  | 30 | Burn | 0.05443 | 0.0295 | 1.848 | 0.1194 | ns |
|  | 30 | Control | 0.03461 | 0.0270 | 1.282 | 0.2664 | ns |
|  | 32 | Amputation | -0.00436 | 0.0273 | -0.159 | 0.9010 | ns |
|  | 32 | Burn | -0.00513 | 0.0295 | -0.174 | 0.9010 | ns |
|  | 32 | Control | 0.00514 | 0.0270 | 0.190 | 0.9010 | ns |
|  | 34 | Amputation | -0.03879 | 0.0273 | -1.419 | 0.2200 | ns |
|  | 34 | Burn | -0.05983 | 0.0295 | -2.031 | 0.0845 | ns |
|  | 34 | Control | -0.02645 | 0.0270 | -0.980 | 0.4133 | ns |
|  | 36 | Amputation | 0.04300 | 0.0273 | 1.573 | 0.1735 | ns |
|  | 36 | Burn | -0.02655 | 0.0295 | -0.901 | 0.4409 | ns |
|  | 36 | Control | -0.00336 | 0.0270 | -0.124 | 0.9010 | ns |
|  | 38 | Amputation | 0.14482 | 0.0273 | 5.298 | <0.0001 | *** |
|  | 38 | Burn | 0.08822 | 0.0295 | 2.994 | 0.0060 | ** |
|  | 38 | Control | 0.04774 | 0.0270 | 1.769 | 0.1290 | ns |
|  | 40 | Amputation | 0.33271 | 0.0273 | 12.172 | <0.0001 | *** |
|  | 40 | Burn | 0.24062 | 0.0295 | 8.167 | <0.0001 | *** |
|  | 40 | Control | 0.24307 | 0.0270 | 9.004 | <0.0001 | *** |
|  | 42 | Amputation | 0.38528 | 0.0274 | 14.040 | <0.0001 | *** |
|  | 42 | Burn | 0.30082 | 0.0295 | 10.211 | <0.0001 | *** |
|  | 42 | Control | 0.30047 | 0.0270 | 11.130 | <0.0001 | *** |

Table S7: Sample sizes in the chronic thermal sensitization assay.

| Treatment | Time | Sample size (n) |
| --- | --- | --- |
| Control | 20 min | 100 |
| Control | 24 h | 96 |
| Control | 96 h | 88 |
| Amputation | 20 min | 100 |
| Amputation | 24 h | 93 |
| Amputation | 96 h | 85 |
| Burn | 20 min | 100 |
| Burn | 24 h | 83 |
| Burn | 96 h | 70 |

3.2 Hyperalgesia

Table S8: Effect of noxious temperatures (38-42 °C) on movement speed. At 24 hours post-anaesthetic, amputated ants showed the steepest temperature response out of the three treatments. Pairwise comparisons of slopes were computed using estimated marginal means (GLMM). Significance levels: ***p < 0.001, *p < 0.05, ns = not significant.

| Time post-anaesthetic | Comparison | Estimate | SE | z-ratio | p-value | Re-sult |
| --- | --- | --- | --- | --- | --- | --- |
| 20 min | Amputation - Burn | 0.099350 | 0.0132 | 7.503 | <0.0001 | *** |
|  | Amputation - Control | -0.013747 | 0.0132 | -1.042 | 0.5505 | ns |
|  | Burn - Control | -0.113097 | 0.0132 | -8.568 | <0.0001 | *** |
| 24 h | Amputation - Burn | 0.133152 | 0.0141 | 9.421 | <0.0001 | *** |
|  | Amputation - Control | 0.039027 | 0.0136 | 2.875 | 0.0113 | * |
|  | Burn - Control | -0.094124 | 0.0140 | -6.727 | <0.0001 | *** |
| 96 h | Amputation - Burn | 0.074554 | 0.0151 | 4.943 | <0.0001 | *** |
|  | Amputation - Control | -0.000535 | 0.0142 | -0.038 | 0.9992 | ns |
|  | Burn - Control | -0.075090 | 0.0149 | -5.028 | <0.0001 | *** |

Table S9: Effect of noxious temperatures (38-42 °C) on proportion of time moving. At 20 minutes, amputated ants showed the steepest temperature response, indicating heightened sensitivity. Pairwise comparisons of slopes were computed using estimated marginal means (GLMM). Significance codes: ns = not significant, ** = p ≤ 0.01, *** = p ≤ 0.001.

| Time post-anaesthetic | Comparison | Estimate | SE | z-ratio | p-value | Result |
| --- | --- | --- | --- | --- | --- | --- |
| 20 min | Amputation - Burn | 0.01049 | 0.00744 | 1.409 | 0.3360 | ns |
|  | Amputation - Control | 0.01851 | 0.00742 | 2.494 | 0.0338 | * |
|  | Burn - Control | 0.00802 | 0.00742 | 1.080 | 0.5262 | ns |
| 24 h | Amputation - Burn | 0.02130 | 0.00795 | 2.680 | 0.0201 | * |
|  | Amputation - Control | 0.00579 | 0.00764 | 0.758 | 0.7289 | ns |
|  | Burn - Control | -0.01552 | 0.00787 | -1.971 | 0.1194 | ns |
| 96 h | Amputation - Burn | 0.00771 | 0.00849 | 0.908 | 0.6352 | ns |
|  | Amputation - Control | -0.00120 | 0.00798 | -0.150 | 0.9876 | ns |
|  | Burn - Control | -0.00891 | 0.00841 | -1.060 | 0.5390 | ns |

3.3 Survival

Table S10: Survival probability in the chronic thermal sensitization assay. The table shows the number of individuals at risk, the number of events, survival estimates and standard errors (SE) for each treatment at different time points. Survival estimates decrease over time, with the burn treatment group showing the lowest survival probability at later time points.

| Time [h] | Treatment | N at Risk | Events | Survival | SE |
| --- | --- | --- | --- | --- | --- |
| 1 | Amputation | 100 | 2 | 0.98 | 0.0140 |
| 24 | Control | 100 | 4 | 0.96 | 0.0196 |
| 24 | Amputation | 98 | 5 | 0.93 | 0.0255 |
| 24 | Burn | 100 | 17 | 0.83 | 0.0376 |
| 48 | Control | 96 | 5 | 0.91 | 0.0286 |
| 48 | Amputation | 93 | 2 | 0.91 | 0.0286 |
| 48 | Burn | 83 | 7 | 0.76 | 0.0427 |
| 72 | Control | 91 | 1 | 0.90 | 0.0300 |
| 72 | Amputation | 91 | 2 | 0.89 | 0.0313 |
| 72 | Burn | 76 | 2 | 0.74 | 0.0439 |
| 96 | Control | 90 | 3 | 0.87 | 0.0336 |
| 96 | Amputation | 89 | 4 | 0.85 | 0.0357 |
| 96 | Burn | 74 | 4 | 0.70 | 0.0458 |

Table S11: Survival analysis. Ants in the burn treatment group exhibited significantly lower survival compared to the control and amputation groups (log-rank tests). Significance levels: **p < 0.01, *p < 0.05, ns = not significant. N = 100 per treatment at time 0.

| Comparison | Treatment | Observed | Expec-ted | Chi-sq | df | p-value | Re-sult |
| --- | --- | --- | --- | --- | --- | --- | --- |
| All groups | Control | 13 | 20.0 |  |  |  |  |
|  | Amputation | 15 | 19.6 |  |  |  |  |
|  | Burn | 30 | 18.4 | 11.6 | 2 | 0.003 | ** |
| Amputation vs Burn | Amputation | 15 | 23.2 |  |  |  |  |
|  | Burn | 30 | 21.8 | 6.4 | 1 | 0.01 | * |
| Amputation vs Control | Control | 13 | 14.1 |  |  |  |  |
|  | Amputation | 15 | 13.9 | 0.2 | 1 | 0.7 | ns |
| Burn vs Control | Control | 13 | 22.4 |  |  |  |  |
|  | Burn | 30 | 20.6 | 8.9 | 1 | 0.003 | ** |
