# Supplementary material for: Chronic pain-like responses revealed by allodynia and hyperalgesia in the ant Lasius niger
Source: BMC Biol. 2026 Nov 6;24:235. doi: 10.1186/s12915-026-02784-4 (PMC13757002; doi:10.1186/s12915-026-02784-4)
Supplement: Supplementary file 2 [file 12915_2026_2784_MOESM2_ESM.docx]

Fig. S1: Average speed while moving. Burn ants consistently exhibited the lowest movement speeds, particularly at higher temperatures. A sharp increase in locomotor activity was observed in all treatments between 34 °C and 40 °C. Ants were acclimated at 25 °C on the hot plate. Treatments not sharing a letter are significantly different from each other at the respective temperature (p < 0.05), based on estimated marginal means with Tukey adjustment for multiple comparisons, derived from a generalized linear mixed model (GLMM). Diamonds represent group means; error bars denote 95 % bootstrapped confidence intervals. Sample sizes of 70-100/treatment, dependent on survival (see Additional file 1: Table S7). For further statistics see html document.

Fig. S2: Proportion of time spent moving. At 20 minutes, control ants moved significantly more than burn-treated ants and more than amputated ants at some temperatures. At 24 hours, differences varied by temperature. By 96 hours, almost no significant group differences remained. Diamonds represent group means; error bars denote 95 % bootstrapped confidence intervals. Letters indicate significant differences (p < 0.05, Tukey-adjusted GLMM EMMs). Ants were acclimated at 25 °C; sample sizes of 70-100/treatment, dependent on survival (see Additional file 1: Table S7). For further statistics see html document.

Fig. S3: Baseline average speed while moving. Movement speed remained stable between 25 °C and 36 °C, with significant increases observed from 38 °C onward, suggesting a behavioural shift consistent with a stress or escape response above 36 °C. Temperatures not sharing a letter differ significantly (p < 0.05). Individual data points are shown as grey dots, diamonds represent group means, error bars denote 95 % bootstrapped confidence intervals. N = 100 ants. For further statistics see html document.

Fig. S4: Survival of the ants in the chronic thermal sensitization assay. Kaplan-Meier survival curves illustrate the probability of survival over time for each treatment group. Ants in the burn treatment group exhibited significantly lower survival compared to the control and amputation groups. Significance levels:  **p < 0.01, *p < 0.05, ns = not significant. N = 100 per treatment at time 0.
